# Exploring the Diet-Gut Microbiota-Epigenetics Crosstalk Relevant to Neonatal Diabetes

**DOI:** 10.3390/genes14051017

**Published:** 2023-04-29

**Authors:** Naser A. Alsharairi

**Affiliations:** Heart, Mind & Body Research Group, Griffith University, Gold Coast, QLD P.O. Box 4222, Australia; naser.alsharairi@gmail.com

**Keywords:** neonatal diabetes, gestational diabetes, gut microbiota, diet, gene expression, epigenetic

## Abstract

Neonatal diabetes (NDM) is a rare monogenic disorder that presents as hyperglycemia during the first six months of life. The link between early-life gut microbiota dysbiosis and susceptibility to NDM remains uncertain. Experimental studies have demonstrated that gestational diabetes mellitus (GDM) could develop into meconium/gut microbiota dysbiosis in newborns, and thus, it is thought to be a mediator in the pathogenesis of NDM. Epigenetic modifications have been considered as potential mechanisms by which the gut microbiota and susceptibility genes interact with the neonatal immune system. Several epigenome-wide association studies have revealed that GDM is associated with neonatal cord blood and/or placental DNA methylation alterations. However, the mechanisms linking diet in GDM with gut microbiota alterations, which may in turn induce the expression of genes linked to NDM, are yet to be unraveled. Therefore, the focus of this review is to highlight the impacts of diet, gut microbiota, and epigenetic crosstalk on altered gene expression in NDM.

## 1. Introduction

Diabetes development in the first months of life is unlikely to be related to Type 1 diabetes (T1D) autoimmunity [1,2]. Neonatal diabetes (NDM), also termed congenital diabetes, is rare and presents in infants up to 6 months of age. It has more than 20 different monogenic causes, for example, mutations in signal transducer and activator of transcription 3 (STAT3) and LPS-responsive beige-like anchor (*LRBA*) genes [3,4]. NDM has been diagnosed in extremely preterm infants [5,6,7,8], particularly those with mutations in chromosome 6q24, GATA binding protein 6 (*GATA6*), and potassium inwardly rectifying channel, subfamily J, member 11 (*KCNJ11*) [7,8]. Infants diagnosed with NDM with no mutation in the *KCNJ11* gene were less likely to have T1D-associated high-risk human leukocyte antigen (HLA) genotypes (DR3-DQ2/X, DR4-DQ8/X, and DR4-DQ8/DR3-DQ2) [1]. Gestational diabetes mellitus (GDM), characterized by increased peripheral insulin resistance (IR), may cause fetal complications, including weight gain, glucose intolerance, and death. However, its effect on NDM remains uncertain [9].

The colonization of the gut with microbes starts prenatally, in utero, after birth, and during breastfeeding. According to the sterile womb hypothesis, microbes are transported from the mother through the lymphatic system or blood stream into the placenta and then translocated to the fetal gut [10,11,12]. The maternal gut microbiota during pregnancy is crucial in shaping the composition of the gut microbiota and immune functions early in life, where diet and other factors (e.g., gestational age, maternal obesity, antibiotic usage) are found to influence the infant gut microbial diversity and richness, which in turn may enhance gut dysbiosis and disease susceptibility later in life [12,13,14,15,16]. Gut microbial dysbiosis in early life plays a significant role in the development of inflammation-related diseases, such as obesity, asthma, inflammatory bowel disease, and necrotizing enterocolitis [17,18,19,20].

The gut microbiota dysbiosis involved in NDM remains unclear. Compelling experimental studies reported that GDM could result in maternal gut and/or neonatal meconium microbiota dysbiosis, characterized by an increased abundance of Actinobacteria and Proteobacteria, *Streptococcus*, *Bacteroides*, *Lachnospiraceae*, *Clostridium*, *Klebsiella*, *Desulfovibrio*, *Rothia*, *Shigella*, *Escherichia*, *Collinsella*, and *Proteus* [21,22,23,24,25]. Diet has been shown to induce gut dysbiosis in women with GDM, along with gut dysbiosis in their newborns [26,27]. However, the exact mechanisms by which diet in GDM alters gut microbiota composition, which may in turn influence gene expression in NDM, are not well understood. A potential hypothesis is that epigenetic mechanisms mediate the effects of diet-microbiota interactions on altering gene expression in NDM. In general, there is a complex interaction between epigenetics, diet, and gut microbiota that can influence gene expression profiles in NDM. Therefore, this review highlights the epigenetic mechanisms mediating the relationships between diet-microbiota interactions and gene expression changes in NDM.

## 2. Methods

A literature search was performed in the PubMed/MEDLINE database and Google Scholar up to February 2023 for studies published in English exploring the impacts of diet, gut microbiota, and epigenetic crosstalk on altering gene expression in NDM. The following keywords were searched: “GDM”, “NDM”, “IR”, “pancreatic β-cells”, “neonates/newborns”, “gut microbiota”, “diet”, “epigenetic”, and “gene expression”. Studies with the main focus on the associations between diet and gut microbiota in GDM and/or their newborns were considered. Human and in vitro studies were considered without study design restrictions.

## 3. An Overview of NDM

NDM is classified into transient (TNDM), permanent (PNDM), or syndrome types, which have expressed significant genetic changes causing persistent hyperglycemia, reduced β-cell mass or replication, delayed pancreatic islet development, and impaired insulin secretion [28]. Autosomal recessive or dominant mutations in the preproinsulin (*INS*) and the ATP-sensitive potassium (K_ATP_) channel (very common to *KCNJ11* or ATP-binding cassette transporter subfamily C member 8, *ABCC8*) are the major genes responsible for TNDM and PNDM. PNDM can also result from autosomal recessive mutations in the glucokinase (*GCK*) and pancreatic and duodenal homeobox l (*PDX1*) genes. Compound heterozygous or homozygous mutations in *GCK* and *PDX1* may cause β-cell glucose sensing impairment and hypoplasia or pancreas agenesis. Insulin or sulfonylurea therapy can often be used for infants with mutations in *KCNJ11*, *ABCC8*, and *INS* [29,30,31,32,33,34,35]. A previous study identified a novel imprinted gene (protein phosphatase 1 regulatory subunit 13 like, *PPP1R13L*) on chromosome 19q13.32 that is hypomethylated in TNDM and associated with the zinc finger protein 57 homolog (*ZFP57*) [36]. TNDM is seen most often in cases of hyperglycemia and intrauterine growth retardation and may cause IR later in life [37]. An experimental study demonstrated that the CC dinucleotide sequence of the human *INS* gene’s active chromatin during pancreas development is mutated in TNDM. The CC dinucleotide mutation also results in disrupted GLI-similar 3 (*GLIS3*)-dependent activation of an episomal *INS* gene [38].

The most common cause of syndrome NDM is autosomal recessive mutations in eukaryotic translation initiation factor 2-α kinase 3 (*EIF2Ak3*) (diabetes associated with renal dysfunction/epiphyseal dysplasia), Solute carrier family 2 member 2 (*SLC2A2*) (diabetes associated with facilitated glucose transporter), Solute carrier family 19, member 2 (*SLC19A2*) (diabetes associated with megaloblastic anemia syndrome), Insulin receptor (*INSR*) (diabetes associated with severe IR), and Forkhead box P3 (*FOXP3*) (diabetes associated with polyendocrinopathy/immunodysregulation) [29,30,31,34,35]. NDM causes insulin deficiency as a result of β-cell destruction or the impaired function of β-cells [2]. Monogenic NDM is associated with growth restriction in utero because of insulin insufficiency that relies on gene mutations in the brain, which may lead to neurodevelopmental disability [39]. There is recent evidence for severe insulin deficiency, increased islet β-cell destruction, and low birthweight and C-peptide levels in infants diagnosed with a high polygenic risk in the first six months of life [40].

## 4. Epigenetic Modifications and NDM Gene Expression Profiles in Neonates Exposed to GDM

Epigenetic changes in several genes involved in GDM are thought to impact newborn metabolic disease susceptibility [41,42,43]. A recent genome-wide methylation analysis identified many enriched pathways for hypo/hyper-differential methylation genes (DMGs) in the placenta and/or the umbilical cord blood of newborns exposed to maternal GDM. The top-ranking pathway enriched in 84 DMGs was the “insulin secretion/IR” pathway [44]. It has been shown that alterations of DNA methylation characterized by significant hypermethylation at two cytosine-phosphate-guanine dinucleotide (CpG) sites and hypomethylation at all CpG sites in adipose tissues of women with GDM and fetal cord blood cells are responsible for reduced adiponectin mRNA expression associated negatively with blood glucose and homeostatic model assessment-IR (HOMA-IR) [45]. This suggests that reduced adipose tissue adiponectin expression may be considered a pathogenic factor in GDM offspring [45]. A study analyzing the DNA methylation profile in the cord blood of newborns exposed to women with GDM has identified 200 differentially methylated loci. Some metabolic disease/T1D-related genes (interleukins 6 and 10; IL-6, IL-10) and pathways enriched by differentially methylated loci were identified. The top metabolically related signaling pathways, including mitogen-activated protein kinase (MAPK), Janus kinase (JAK), phosphatidylinositol-3 kinase (PI3K), and STAT3, were identified [46].

Epigenetics is considered a key mechanism that affects glucose metabolism genes involved in GDM, and their dysregulation leads to differential DNA methylation of the tribbles homolog 1 (*TRIB1*) gene and vasoactive intestinal peptide receptor (VIPR1) in the placenta and fetal cord blood [47]. VIPR1 is highly expressed in pancreatic β-cells, which may activate adenylate cyclase and insulin secretion by increasing intracellular cyclic AMP (cAMP) production, which in turn stimulates protein kinase A (PKA) and increases optimal calcium influx. Genetic deletion of VIPR1 could lead to glucose intolerance [48]. The *TRIB1* gene, mapped to chromosome 8q24 [49], was found to be influenced by GDM exposure in the umbilical vein endothelial cells of newborns [50]. An experimental study showed that the *TRIB1* gene was associated with pro-inflammatory gene cyclooxygenase-2 (*COX-2*) overexpression by the action of regulated early growth response gene-1 (*EGR-1*), which resulted in increased glucose levels in small for gestational age neonate-derived mesenchymal stem cells [51]. In pancreatic islet β-cells, *COX-2* expression was associated with downregulation of *PDX1*-related NDM by increasing the IL-1β autostimulation [52].

The placental tissue of women with GDM induces pro-inflammatory gene expression (tumor necrosis factor-α, *TNF-α*) that dysregulates insulin signaling and reduces insulin secretion from β-cells under the condition of hyperglycemia [53]. Placental GDM is the major secretion site of growth hormones (e.g., insulin-like growth factor, IGF) that play a role in stimulating pro-inflammatory cytokine production by activation of inflammatory pathways, such as PI3K [53], which increases glucose levels in the fetal cord blood and may result in NDM. The results of previous studies suggest an association between the GDM intrauterine environment and placental DNA methylation [53,54]. Higher placental DNA methylation of the PPAR-γ coactivator-1-α (*PGC1α*) gene was associated with IR and insulin secretion in women with GDM [54]. High maternal glucose levels were reported to be associated with placental DNA methylation changes to the *PGC1α* gene on chromosome 4p15.1 in GDM, suggesting that *PGC-1α* disturbs placental functions, which may increase the risk of diabetes in offspring [55]. *PGC1α* mRNA expression in human adipocytes has been linked to IR markers. Patients with IR and visceral obesity have demonstrated reduced *PGC1α* mRNA expression in adipose tissue, which leads to increased adiponectin and IL-6 serum levels [56]. In one longitudinal study, maternal GDM was shown to induce high DNA methylation variations at the *PGC1α* gene locus, and such variations may mediate the impact of GDM on increasing fetal cord blood glucose levels [57]. *PGC1α* mRNA expression in both adipose and placenta tissue of GDM women has an impact on glucose and lipid homeostasis by increasing adiponectin and low density lipoprotein (LDL) cholesterol levels and decreasing tryglycerides and glucose levels [58]. Levels of *PGC1α* and *PDX1* were reported to be reduced in placental tissue of women with GDM, which may lead to abnormal glucose metabolism in newborns [59]. Upregulating *PGC1α* activity in the brain and lungs of preterm infants has a significant role in activating the transcription factors implicated in mitochondrial biogenesis and increasing mitochondrial antioxidant enzymes, which in turn may reduce inflammation and oxidative stress (OS) by downregulating pro-inflammatory cytokines and chemokines [60]. Overexpression of the forkhead box O 1 (FoxO1) in pancreatic β-cells regulated by glucagon-like peptide 1 (GLP-1) stimulation results in inhibited *PGC1α* and its target gene, *PDX1*-related NDM [61].

The imprinted mesoderm-specific transcript (*MEST*) gene showed significant DNA methylation at five CpG sites in the cord blood of GDM newborns, which in turn led to decreased *MEST* methylation, thus influencing obesity and diabetes susceptibility [62]. A large-scale, genome-wide study has identified a total of 4485 hypermethylated and hypomethylated CpG sites in 2198 differentially methylated genes (e.g., *MEST*) enriched in the T1D pathway in the cord blood of infants born to GDM women [63]. *MEST*, located on chromosome 7q32, a gene belonging to a cluster of carboxypeptidase A (CPA) genes, has been implicated in postnatal and intrauterine growth restriction related to congenital Silver-Russell syndrome (SRS) [64]. Evidence suggests that paternal inherited H19/IGF2:IG-DMR deletions interfering with *ZFP57* involved in NDM may result in SRS [65]. A case report has shown that maternal inheritance at chromosomes 2, 8, and 21 in the region of PLAG1 like zinc finger 1 (*PLAG1*)-associated NDM is responsible for SRS [66].

A pilot study has identified differentially methylated regions of POU class 2 homeobox 1 (*POU2F1*), paraoxonase 1 (*PON1*), and NF-E2 related factor 2 (*NRF2*) in the cord blood of newborns of GDM women [67]. *POU2F1* is found on chromosome 1q24, a locus with evidence of strong linkage disequilibrium for its relationship to type 2 diabetes (T2D) [68]. Treatment of pancreatic β-cells with hydrogen peroxide (H_2_O_2_) results in enhanced *POU2F1* activity as well as other inflammatory signaling pathway activation, such as c-jun N-terminal kinase (JNK) and DNA-dependent protein kinase (DNA-PK) [69], which in turn may increase IR and diabetes susceptibility. Methylation for the *PON1* gene in mothers, which is localized on chromosome 7q21-22 [70], has been observed in children of mothers exposed to adverse life events, which coincides with the presence of the *ZFP57* gene implicated in NDM [71]. The Q192R polymorphism of the *PON1* gene was reported to increase GDM susceptibility, which could be a marker for IR [72]. High *PON1* levels and *PON1* lactonase activity were associated with increased OS, which causes alterations of the glycolipid metabolic profiles in infants born to GDM women [73]. Neonates demonstrate increased free *PON1*, decreased *PON1* lactonase activity, and different PON1 distribution in the high-density lipoprotein (HDL) subclasses in cord blood. *PON1* lactonase activity was observed to be lower in the large HDL group than in the small HDL group [74]. Impaired *NRF2* activity was reported to increase IR and OS associated with diabetes, which can contribute to decreased antioxidant enzyme activity in pancreatic β-cells [75]. GDM contributes to fetal *NRF2*-mediated antioxidant signaling dysregulation in fetal endothelial cells by increasing OS, protein carbonylation, and mitochondrial reactive oxygen species (ROS) generation [76]. *NRF2* has been shown to restore *PDX1* levels in pancreatic β-cells by reducing OS-mediated JNK-dependent FOXO1 activation [77]. A candidate gene study showed that downregulation of *PDX1* mRNA expression in the placentas of women with GDM resulted in increased blood glucose levels in fetal cord blood [59].

A recent meta-analysis of epigenome-wide association studies showed that maternal hyperglycemia during pregnancy is associated with reduced offspring DNA methylation at two CpG sites located in the thioredoxin interacting protein (*TXNIP*) gene [78]. Overexpression of *TXNIP*, also termed α-arrestin, in pancreatic β-cells increases glucose levels by binding to and suppressing the antioxidant protein thioredoxin (TXN), which may lead to impaired activity of the angiogenic cytokine vascular endothelial growth factor (VEGF), increased ROS expression, induction of apoptosis, and decreased insulin production [79,80]. Overexpression of *TXNIP* mRNA in the placenta increases ROS production and mitochondrial dysfunction as a result of decreasing TXN expression levels [81]. In one study, *TXNIP* mRNA expression was reported to increase in GDM women but not in neonates. On the other hand, TXN mRNA expression in the placenta was high. The thioredoxin (TXN)/TXNIP ratio increases in the placenta and neonatal cord blood of GDM women, concurrent with increased expression of nuclear factor-kappa B (NF-_k_B), as well as STAT3 and its target protein suppressor of cytokine signaling 3 (SOCS3) [82]. This indicates that TXN expression in the placenta may exert a protective role in protecting the newborn from oxidative effects. Another study has linked NDM with an aberrant activation of STAT3, which leads to pancreatic β-cell dysfunction and reduced insulin expression [83]. 

**Figure 1:**
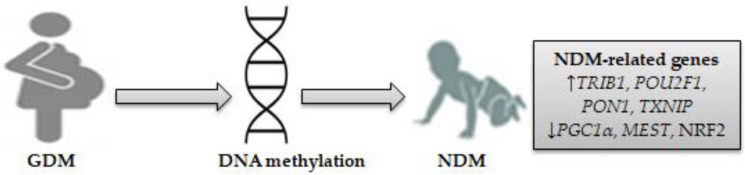
DNA methylation as a mechanism for NDM-related genes in neonates exposed to GDM. Seven differentially expressed genes involved in NDM were identified, consisting of four upregulated genes (*TRIB1*, *POU2F1*, *PON1*, *TXNIP*) and three downregulated genes (*PGC1α*, *MEST*, and *NRF2*). (↓) decrease, (↑) increase.

These findings suggest that exposure to GDM causes alterations in placental and/or fetal cord blood DNA methylation, which in turn may impact fetal glucose metabolism genes involved in NDM. Differentially expressed genes might be enriched in the PI3K, STAT3, JAK, and MAPK signaling pathways and other inflammatory genes, such as *COX-2*, IL-6, and IL-10. On this basis, seven genes related to NDM were identified: *TRIB1*, *PGC1α*, *MEST*, *POU2F1*, *PON1*, *NRF2*, and *TXNIP*. Figure 1 shows NDM-related gene-specific DNA methylation in neonates exposed to GDM.

## 5. Diet, Gut Microbiota, and Epigenetic Crosstalk Alters Gene Expression in NDM

Gut microbiota, as one environmental factor, may modulate epigenetic mechanisms through DNA methylation alterations in response to diet, which may affect glucose metabolism genes involved in NDM. This section focuses on the crosstalk between diet, gut microbiota, and microbial-derived metabolites (short-chain fatty acids, SCFAs), considered epigenetic modifiers, which could in turn influence gene expression profiles in NDM.

### 5.1. Diet Alters Gut Microbiota in GDM and NDM

Diet is considered the key environmental factor contributing to the pathogenesis of islet autoimmunity by modulating the gut microbiota, which may lead to dysbiosis, characterized by increased intestinal inflammation, permeability, and reduced mucosal barrier integrity [84]. The intestinal permeability as a result of increased levels of tight junction (TJ)-related proteins (e.g., zonulin, occludin, and claudin-2) influenced by microbial colonization could trigger autoimmunity by allowing microbial or dietary antigens to be transferred to the circulation, leading to pancreatic β-cell destruction that can contribute to intestinal inflammation [85].

A few studies have shown that diet may influence gut microbiota in women with GDM and their infants. A prospective study of GDM women showed that dietary fat intake (polyunsaturated fatty acid, PUFA; saturated fatty acid, SFA) is associated with a higher abundance of gut Bacteroidetes *Alistipes*, while dietary fiber intake is associated with a higher abundance of gut Firmicute *Roseburia* [26]. In another prospective study, GDM women consuming a complex carbohydrate (CHO) and low-fat diet (choice diet) showed a higher abundance of gut *Bifidobacterium* spp. (particularly *B. adolescentis*) than those consuming a high-fat diet (conventional diet). The study also revealed a high relative abundance of *Prevotella copri* (*P. copri*), *Enterobacter cloacae* (*E. cloacae*), *Enterococcus faecalis* (*E. faecalis*), and *Bacteroides* in the guts of infants aged ≤ 4 months born to women on a conventional diet [27]. This suggests that diet in women with GDM is associated with the composition of the neonatal gut microbiota.

### 5.2. Gut Microbiota and Its Metabolites Alter Gene Expression in NDM

The generation of metabolites produced by gut microbiota in response to diet, such as SCFAs, could be considered an epigenetic mechanism that may influence gene expression in NDM. SCFAs and butyrate in particular have been shown to alleviate inflammation in infant intestinal epithelial cells (IECs) through their ability to inhibit histone deacetylases (HDACs) and activate G-protein coupled/free fatty acid receptors (GPRs/FFAs) in dendritic cells (DCs), which promote differentiation of FOXP3^+^ regulatory T (T_reg_) cells. This result in inhibited NF-κB activation, lipopolysaccharide (LPS)-induced pro-inflammatory cytokine and chemokine secretion, and induced PPARγ-dependent pathway activation [17,18,19,20]. SCFAs enhance intestinal barrier integrity by reducing blood glucose levels, increasing protective GLP-1 production, and improving IR by activating NF-κB and STAT3 [86], identified as an NDM-related gene [82,83]. In human monocyte-macrophage cells, SCFAs were shown to inhibit the nucleotide-binding oligomerization domain-like receptor family pyrin domain containing 3 (NLRP3) inflammasome by increasing *Nrf2*-mediated TXN1, which reacts with *TXNIP* implicated in NDM, resulting in a decreased expression of *TNF-α* and IL-1β [87]. SCFAs were also reported to improve intestinal barrier function by reducing LPS-induced *TXNIP*-mediated NLRP3 activation and TJ-related proteins (zonulin and occludin) [88]. Butyrate has been found to increase *Nrf2* mRNA levels and inhibit protein 53 (p53) mRNA levels in IECs [89].

Diets rich in dietary fiber, complex CHO, and/or dietary fat induce changes in the GDM and their newborn microbiome, with increases in the Gram-positive Actinobacteria (*Bifidobacterium*) and Firmicutes (*Roseburia*, *E. faecalis*) and the Gram-negative Bacteroidetes (*Bacteroides*, *P. copri*, *Alistipes*) and Enterobacteriaceae (*Enterobacter cloacae*, *E. cloacae*).

#### 5.2.1. Actinobacteria

A few studies have shown that *Bifidobacterium* spp. (including *B. adolescentis*) are transferred from mothers to their neonates [90,91,92,93]. In one study, *Bifidobacterium* spp. were found to be enriched in the gut of breastfed infants born to GDM women [94], while in another study, *Bifidobacterium* spp. were depleted in the gut of GDM women [95]. In full-term infants born to GDM women, *Bifidobacterium* spp. was associated with reduced fecal propionate levels [96]. *Bifidobacterium* spp. are able to utilize human milk oligosaccharides (HMOs) (e.g., fructo-oligosaccharide and galacto-oligosaccharide) through glycoside hydrolase (GHs)-related degradation enzymes, resulting in increased production of butyrate, propionate, and acetate [20]. *Bifidobacterium* spp. are reported to ferment resistant starch in the newborn gut prior to the weaning period, which results in the production of SCFAs [97]. Probiotic-supplemented formulas increase *Bifidobacterium* spp. abundance in preterm gut microbiota [20]. Adherence to a very low-calorie ketogenic diet (VLCKD) during pregnancy, characterized by low CHO, moderate protein, and high fat intake, influences the infant gut microbiota composition and leads to an increase in the abundance of *Bifidobacterium* spp., which exerts protective effects against inflammation-related diseases by inhibiting several signaling pathways [17,18,19,20]. Butyrate and acetate-producing *B. lactis* spp. 420 resulted in reduced *COX-2* expression in the enterocyte-like cell line Caco-2 [98]. In vitro, treatment of macrophage RAW264 cells with *B. adolescentis* and quercetin induced anti-inflammatory effects by reducing LPS-stimulated *TNF-α* and IL-1β production and inducible nitric oxide synthase (iNOS) and *COX-2* expression [99]. Thus, *B. adolescentis* may exert its anti-inflammatory effects on NDM by reducing *TRIB1* gene-mediated *COX-2* expression.

#### 5.2.2. Firnicutes

There is evidence to support vertical transmission of *Roseburia* from mother to infant by breastfeeding [100]. Gut dysbiosis in GDM women is characterized by a reduced abundance of *Roseburia*, which was associated with increased blood glucose levels and IR [101]. The VLCKD could be considered a contributing factor influencing the gut butyrate-producing *Roseburia intestinalis* (*R. intestinalis*) in infants and children, which exerts anti-inflammatory effects in IECs as indicated by increasing transforming growth factor-β (TGF-β) and inhibiting LPS-induced *TNF-α* and IL-17 secretion [19]. IL-17 is involved in the dysregulation of insulin production from β-cells by activation of NF-κB and STAT1 signaling pathways, resulting in increased interferon (IFN)γ and IL-1β-induced *TNF-α* production [53,102]. *R. intestinalis* and *R. hominis* are shown to upregulate the *PGC1α* gene in IECs [103], which is reported to be downregulated in adipose and placental tissue of GDM women, resulting in increased IR markers and fetal cord blood glucose levels [57,58]. It can be suggested that *Roseburia* spp. may exert anti-inflammatory effects in the IECs of GDM neonates, providing a potential therapeutic role for NDM by upregulating the *PGC1α* gene.

A previous study supports the notion of vertical transmission of *Enterococcus* spp. from the mother to her infant by vaginal delivery [104]. *Enterococcus* forms L(+)-lactic acid as the key end product of sugar fermentation [105]. *Enterococcus* is able to ferment resistant starch in pre-weaned or weaning infants, which in turn produces butyrate, propionate, and acetate [97]. Early probiotic supplementation with *B. breve* M-16V resulted in increased fecal *Enterococcus* proportions in preterm infants [20]. The presence of *E. faecalis* was shown to decrease blood glucose levels in the human IEC line Caco-2 after feeding with a diet containing glucose or sucrose [106]. *E. faecalis* induces anti-inflammatory effects in infant IECs through its ability to inhibit tumor-receptor associated factors by inhibiting JNK and MAPK signaling pathways [17]. In neonatal IEC, *E. faecalis* was reported to activate anti-inflammatory cytokine IL-10 production and DNA binding of the transcription factor *PGC1α* gene [107], which is known to regulate blood glucose levels in NDM. Thus, *E. faecalis* may exert a protective effect against NDM due to its ability to promote the *PGC1α* gene.

#### 5.2.3. Bacteroidetes

Evidence from strain-level vaginal microbial detection using a metagenomic approach confirms the mother and the newborn shared *Bacteroides* spp. as a result of vertical transmission [91,92,93,108]. Recent studies have shown an increased abundance of gut *Bacteroides* spp. in GDM women and/or their newborns [109,110,111]. *Bacteroides* contain several GHs involved in mucin glycan degradation that maintain gut mucosal barrier integrity [112,113]. SCFAs are largely produced by *Bacteroides* in weaned infants’ feces in response to resistant starch fermentation [97], through the acetyl-CoA and succinate pathways [114]. *Bacteroides* spp., including *B. thetaiotaomicron*, *B. vulgatus*, and *B. fragilis*, are SCFA-producing bacteria that may exert anti-inflammatory effects in the inflamed IECs in infants in response to adherence to the VLCKD during pregnancy, resulting in reduced pro-inflammatory cytokine expression by epigenetic mechanisms related to DNA methylation and non-coding RNAs (lncRNA) [17,18,19,20]. In one study, *B. vulgatus* was shown to increase plasma IL-6 and IR in T2D patients [115], while in another study, unclassified *Bacteroides* were reduced in GDM women [110]. Evidence from an experimental study showed that *B. fragilis* promotes the Sulfiredoxin-1 (Srx-1) enzyme responsible for decreasing peroxiredoxin hyperoxidation by activating *Nrf2* siRNA-related NDM in IECs treated with enterotoxin-induced OS and DNA damage [116]. This suggests that *B. fragilis* may protect against NDM by reducing oxidative damage in IECs through upregulation of the *NRF2* gene.

There is evidence suggesting a vertical transmission of fecal *Alistipes* from mothers to infants following vaginal birth [93]. The genus *Alistipes* belongs to the GH family, which is essential for mucin degradation in the gut [113]. Compared to a plant-based diet, an animal-based diet had a greater influence on gut microbiota alterations by increasing the abundance of SCFA-producing *Alistipes* [117]. Decreases in *Alistipes* abundance may lead to gut dysbiosis and contribute to GDM. The results of previous studies demonstrated an increased abundance of *Alistipes* spp. in the gut microbiota of healthy pregnant women compared to GDM women, which was negatively associated with glucose tolerance and increased sensitivity to C-reactive proteins [95,118,119]. *Alistipes* was associated with reduced pancreatic exocrine dysfunction and mucin degradation in T1D patients with islet autoimmunity [120]. *Alistipes* exerts immunomodulatory activity in human peripheral blood mononuclear cells by inhibiting toll-like receptor 4 (TLR-4)-dependent IL-6, IL-1β, and TNF-α production elicited by *Escherichia coli* (*E. coli*) LPS [121]. This suggests that *Alistipes* may exert anti-inflammatory effects in GDM through their ability to enhance gut mucosal barrier function. An in vitro experimental study found an increase in the colonization of diverse gut microbiota, including *Alistipes*, after consumption of anthocyanin-rich fruit juice, which in turn reduced the basal level of *NRF2* gene transcription in peripheral blood lymphocytes [122]. Short-term administration of *COX-2* inhibitor celecoxib increases gut abundance of butyrate-producing *Alistipes* in vitro, resulting in decreased production of IL-8 and C-X-C motif chemokine ligand (CXCL-16) [123]. It can be suggested that *Alistipes* may exert immune-regulatory and anti-inflammatory effects on the neonatal gut through downregulating *TRIB1* gene-mediated *COX-2* expression, which contributes to a decreased risk of NDM.

A few studies have revealed a low abundance of *Prevotella* in the guts of GDM women and their infants [22,124]. *Prevotella* was shown to reduce fecal levels of branched SCFAs (isobutyrat and isovalerate) in GDM women [124] and acetate in neonates born to GDM women [96], suggesting that *Prevotella* may increase inflammatory activities and influence risk for GDM and NDM, in part by reducing SCFA levels. *Prevotella* was found to promote inflammation and reduce TJ integrity, as demonstrated by a study stimulating IECs to produce pro-inflammatory cytokines (e.g., IL-6, IL-8) [125]. Among *Prevotella* spp., *P. copri* was associated with HOMA-IR [126]. *P. copri* increases LPS-induced IL-6 production associated with IR and low-grade inflammation [115]. An increased abundance of *P. copri* was associated with adherence to an omnivorous dietary regimen, characterized by low levels of lipid metabolism-related miRNA expression [127], which is regulated by the activity of *Nrf2*-related NDM [128]. This suggests that *P. copri* drives unstable epigenetic changes in the gut of GDM newborns, which may increase NDM susceptibility.

#### 5.2.4. Enterobacteriaceae

Few studies have confirmed the mother-to-infant transmission of *Enterobacter* spp. (*E. cloacae* and *E. aerogenes)* by vaginal delivery [91,108], which act as bacterial pathogens causing nosocomial infections [129]. Enterobacteriaceae, particularly *E. cloacae*, are dominant with a high relative abundance in the gut microbiota of GDM women [95]. In vitro, *E. cloacae* abundance is associated with the long-term glucose marker hemoglobin A1c (HbA1c), LPS binding protein (LBP), and C-reactive peptide (CRP). It also induces pancreatic β-cell inflammation through downregulation of the *PDX1* gene, which coincides with an activation of *TXNIP*-mediated NLRP3 involved in NDM, resulting in increased pro-inflammatory cytokines TNF-α, IL-1β, and IL-6 [130]. This suggests that *E. cloacae* may induce changes in the gene patterns that coincide with increasing pro-inflammatory effects in the neonate’s gut, increasing NDM risk.

**Figure 2:**
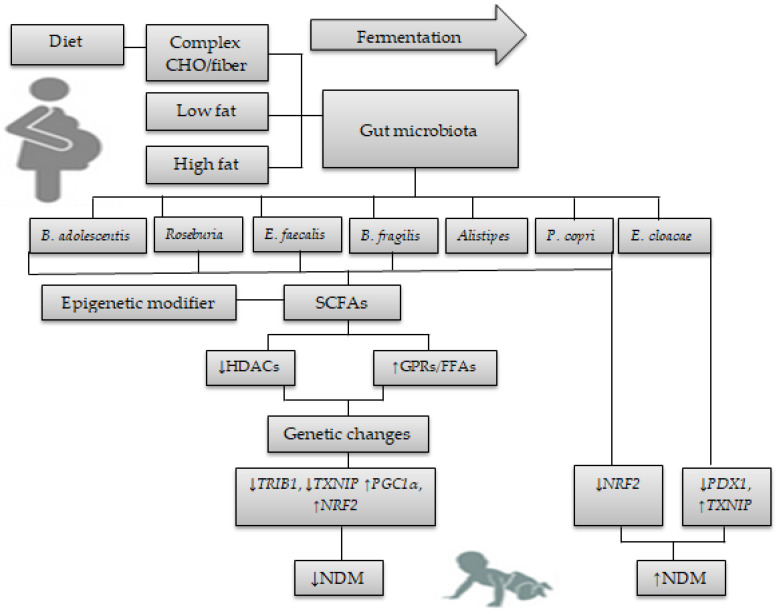
The diet-gut microbiota-epigenetics crosstalk relevant to gene expression alterations in NDM. Maternal diet in GDM (complex CHO/fiber, low/high fat) influences the newborn’s gut microbiota-derived SCFAs. SCFAs produced by *B. adolescentis*, *Roseburia*, *E. faecalis*, *B. fragilis*, and *Alistipes* may act as epigenetic modifiers by inhibiting HDACs and activating GPRs and FFAs, thereby reducing the risk of NDM by increasing gene expression levels of *PGC1α* and *NRF2* and decreasing gene expression levels of *TRIB1* and *TXNIP*. An increased abundance of *P. copri* was associated with reduced fecal levels of SCFAs, which drive unstable genetic changes in the offspring’s gut by reducing *NRF2* gene expression. An increased abundance of *E. cloacae* was associated with an increased gene expression level of *TXNIP* and a decreased gene expression level of *PDX1*. (↓) decrease, (↑) increase.

Figure 2 shows the interplay between diet, gut microbiota, and epigenetics relevant to NDM.

## 6. Conclusions

NDM is monogenic in etiology, with infants who have autosomal recessive or dominant mutations in genes responsible for TNDM, PNDM, and syndrome types. Epigenetics is implicated in NDM susceptibility, and previous studies suggest that the DNA methylation in the cord blood of newborns exposed to GDM is altered, which in turn may impact glucose metabolism genes. NDM is influenced by a comprehensive set of genes. This review identifies seven genes showing significant expression alterations in response to GDM exposure. These include four upregulated genes (*TRIB1*, *POU2F1*, *PON1*, and *TXNIP*) and three downregulated genes (*PGC1α*, *MEST*, and *NRF2*) with NDM.

Few studies have confirmed the potential for gut microbiota to be transmitted from mother to newborn. Gut microbiota dysbiosis in NDM remains largely unknown. GDM is associated with gut microbiota dysbiosis in newborns. Diet and gut microbiota dysbiosis in GDM may be a potential predictive biomarker of NDM. SCFAs are considered epigenome modifiers that alleviate intestinal inflammation and affect the expression of NDM-related genes. 

Maternal diet in GDM was associated with alterations in gene expression in NDM. Interactions between complex CHO/low-fat diets and gut microbiota-derived SCFAs, such as *Bifidobacterium* and *Roseburia*, induce alterations in specific genes involved in the protective effects on NDM by downregulating the *TRIB1* gene and upregulating the *PGC1α* gene. A high-fat diet increases the abundance of *P. copri* and *E. cloacae*, which may cause unstable genetic alterations in the neonate’s gut with implications for NDM. The *NRF2* and *PDX1* genes were downregulated, while the *TXNIP* gene was upregulated with a high-fat diet. Increased levels of *Bacteroides*, *E. faecalis*, and *Alistipes* in response to a high-fat diet may have a protective role against NDM, which results in upregulation of the *NRF2* and *PGC1α* genes and downregulation of the *TRIB1* gene. These bacteria have the ability to produce SCFAs, but further studies are needed to confirm their effects on reducing NDM. The epigenetic mechanisms by which Bacteroidetes *P. copri* and *E. cloacae* might be altering gene expression in NDM need to be fully elucidated in further studies. Further studies are also needed to explore the mechanisms for the pro- and anti-inflammatory effects of commensal and pathogenic bacteria on susceptibility to NDM-related genes in response to maternal diet in GDM.

## Data Availability

Not applicable.

## Abbreviations

ABCC8	ATP-binding cassette transporter subfamily C member 8
cAMP	Cyclic AMP
CHO	Carbohydrate
COX-2	Cyclooxygenase-2
CPA	Carboxypeptidase A
CpG	Cytosine-phosphate-guanine dinucleotides
CRP	C-reactive peptide
CXCL	C-X-C motif chemokine ligand
DCs	Dendritic cells
DMGs	Differential methylation genes
DNA-PK	DNA-dependent protein kinase
EGR-1	Early Growth Response Gene-1
EIF2AK3	Eukaryotic translation initiation factor 2-α kinase 3
FFAs	Free fatty acid receptors
FoxO1	Forkhead box O1
FoxP3	Forkhead box P3
GATA6	GATA binding protein 6
GCK	Glucokinase
GDM	Gestational diabetes
GHs	Glycoside hydrolases
GLIS3	GLI-similar 3
GLP-1	Glucagon-like peptide 1
GPRs	Activate G-protein coupled receptors
H_2_O_2_	Hydrogen peroxide
HbA1c	Hemoglobin A1c
HDACs	Histone deacetylases
HDL	High density lipoprotein
HLA	Human leukocyte antigen
HMOs	Human milk oligosaccharides
HOMA-IR	Homeostatic model assessment of insulin resistance
IECs	Intestinal epithelial cells
IGF	Insulin-like growth factor
IL	Interleukins
IFN-γ	Interferon-γ
lncRNA	Non-coding RNAs
INS	Preproinsulin
INSR	Insulin receptor
IR	Insulin resistance
JAK	Janus kinase
JNK	c-jun N-terminal kinase
K_ATP_	ATP-sensitive potassium
KCNJ11	Potassium inwardly rectifying channel, subfamily J, member 11
LBP	LPS binding protein
LDL	Low density lipoprotein
LPS	Lipopolysaccharide
LRBA	LPS-responsive beige-like anchor
MAPK	Mitogen-activated protein kinase
MEST	Mesoderm-specific transcript
NDM	Neonatal diabetes
NF-_k_B	Nuclear factor-kappa B
NLRP3	Nucleotide-binding oligomerization domain-like receptor family pyrin domain containing 3
iNOS	Nitric oxide synthase
NRF2	NF-E2 related factor 2
OS	Oxidative stress
P53	Protein 53
PDX1	Pancreatic and duodenal homeobox l
PGC1α	PPAR-γ coactivator-1-α
PI3K	Phosphatidylinositol-3 kinase
PKA PLAG1	Protein kinase A PLAG1 like zinc finger 1
PNDM	Permanent neonatal diabetes
PON1	Paraoxonase 1
POU2F1 PPP1R13L	POU class 2 homeobox 1 protein phosphatase 1 regulatory subunit 13 like
PUFA	Polyunsaturated fatty acid
ROS	Reactive oxygen species
SCFAs	Short-chain fatty acids
SFA	Saturated fatty acid
SLC2A2	Solute carrier family 2 member 2
SLC19A2	Solute carrier family 19, member 2
SOCS3	Suppressor of cytokine signaling 3
SRS	Silver-Russell syndrome
Srx-1	Sulfiredoxin-1
STAT3	Signal transducer and activator of transcription 3
T1D	Type 1 diabetes
T2D	Type 2 diabetes
TGF-β	Transforming growth factor-β
TJ	Tight junction
TLR	Toll like receptor
TNDM	Transient neonatal diabetes
TNF-α	Tumor necrosis factor-α
T_reg_	Regulatory T
TRIB1	Tribbles homolog 1
TXN	Thioredoxin
TXNIP	Thioredoxin interacting protein
VEGF	Vascular endothelial growth factor
VIPR1	Vasoactive intestinal peptide receptor
VLCKD	Very low-calorie ketogenic diet
ZEP57	Zinc finger protein 57 homolog

## References

[1] Edghill E.L., Dix R.J., Flanagan S.E., Bingley P.J., Hattersley A.T., Ellard S., Gillespie K.M. (2006). HLA genotyping supports a nonautoimmune etiology in patients diagnosed with diabetes under the age of 6 months. Diabetes.

[2] Rubio-Cabezas O., Ellard S. (2013). Diabetes mellitus in neonates and infants: Genetic heterogeneity, clinical approach to diagnosis, and therapeutic options. Horm. Res. Paediatr..

[3] Flanagan S.E., Haapaniemi E., Russell M.A., Caswell R., Allen H.L., De Franco E., McDonald T.J., Rajala H., Ramelius A., Barton J. (2014). Activating germline mutations in STAT3 cause early-onset multi-organ autoimmune disease. Nat. Genet..

[4] Johnson M.B., De Franco E., Allen H.L., Senani A.A., Elbarbary N., Siklar Z., Berberoglu M., Imane Z., Haghighi A., Razavi Z. (2017). Recessively inherited *LRBA* mutations cause autoimmunity presenting as neonatal diabetes. Diabetes.

[5] Nishimaki S., Yukawa T., Makita Y., Honda H., Kikuchi N., Minamisawa S., Yokota S. (2008). Transient neonatal diabetes mellitus in extremely preterm infant. Arch. Dis. Child.-Fetal Neonatal Ed..

[6] Busiah K., Auger J., Fauret-Amsellem A.-L., Dahan S., Pouvreau N., Cavé H., Polak M., Mitanchez D. (2015). Differentiating transient idiopathic hyperglycaemia and neonatal diabetes mellitus in preterm infants. Horm. Res. Paediatr..

[7] Siklar Z., Ellard S., Okulu E., Berberoğlu M., Young E., Erdeve S.S., Mungan I.A., Hacihamdioğlu B., Erdeve O., Arsan S. (2011). Transient neonatal diabetes with two novel mutations in the KCNJ11 gene and response to sulfonylurea treatment in a preterm infant. J. Pediatr. Endocrinol. Metab..

[8] Besser R.E.J., Flanagan S.E., Mackay D.G.J., Temple I.K., Shepherd M.H., Shields B.M., Ellard S., Hattersley A.T. (2016). Prematurity and genetic testing for neonatal diabetes. Pediatrics.

[9] Bukhari I., Iqbal F., Thorne R.F. (2022). Editorial: Relationship between gestational and neonatal diabetes mellitus. Front. Endocrinol..

[10] Perez-Muñoz M.E., Arrieta M.C., Ramer-Tait A.E., Walter J. (2017). A critical assessment of the “sterile womb” and “in utero colonization” hypotheses: Implications for research on the pioneer infant microbiome. Microbiome.

[11] Walker R.W., Clemente J.C., Peter I., Loos R.J.F. (2017). The prenatal gut microbiome: Are we colonized with bacteria in utero?. Pediatr. Obes..

[12] Senn V., Bassler D., Choudhury R., Scholkmann F., Righini-Grunder F., Vuille-dit-Bille R.N., Restin T. (2020). Microbial colonization from the fetus to early childhood—A comprehensive review. Front. Cell Infect. Microbiol..

[13] Alsharairi N.A. (2020). The infant gut microbiota and risk of asthma: The effect of maternal nutrition during pregnancy and lactation. Microorganisms.

[14] Chu D.M., Meyer K.M., Prince A.L., Aagaard K.M. (2016). Impact of maternal nutrition in pregnancy and lactation on offspring gut microbial composition and function. Gut Microbes.

[15] Nuriel-Ohayon M., Neuman H., Koren O. (2016). Microbial changes during pregnancy, birth, and infancy. Front. Microbiol..

[16] Jeong S. (2022). Factors influencing development of the infant microbiota: From prenatal period to early infancy. Clin. Exp. Pediatr..

[17] Alsharairi N.A. (2020). The role of short-chain fatty acids in the interplay between a very low-calorie ketogenic diet and the infant gut microbiota and its therapeutic implications for reducing asthma. Int. J. Mol. Sci..

[18] Alsharairi N.A. (2021). The role of short-chain fatty acids in mediating very low-calorie ketogenic diet-infant gut microbiota relationships and its therapeutic potential in obesity. Nutrients.

[19] Alsharairi N.A. (2022). The therapeutic role of short-chain fatty acids mediated very low-calorie ketogenic diet-gut microbiota relationships in paediatric inflammatory bowel diseases. Nutrients.

[20] Alsharairi N.A. (2023). Therapeutic potential of gut microbiota and its metabolite short-chain fatty acids in neonatal necrotizing enterocolitis. Life.

[21] Hu J., Nomura Y., Bashir A., Fernandez-Hernandez H., Itzkowitz S., Pei Z., Stone J., Loudon H., Peter I. (2013). Diversified microbiota of meconium is affected by maternal diabetes status. PLoS ONE.

[22] Su M., Nie Y., Shao R., Duan S., Jiang Y., Wang M., Xing Z., Sun Q., Liu X., Xu W. (2018). Diversified gut microbiota in newborns of mothers with gestational diabetes mellitus. PLoS ONE.

[23] Wang J., Zheng J., Shi W., Du N., Xu X., Zhang Y., Ji P., Zhang F., Jia Z., Wang Y. (2018). Dysbiosis of maternal and neonatal microbiota associated with gestational diabetes mellitus. Gut.

[24] Hasain Z., Mokhtar N.M., Kamaruddin N.A., Ismail N.A.M., Razalli N.H., Gnanou J.V., Ali R.A.R. (2020). Gut microbiota and gestational diabetes mellitus: A review of host-gut microbiota interactions and their therapeutic potential. Front. Cell Infect. Microbiol..

[25] Chen T., Qin Y., Chen M., Zhang Y., Wang X., Dong T., Chen G., Sun X., Lu T., White R.A. (2021). Gestational diabetes mellitus is associated with the neonatal gut microbiota and metabolome. BMC Med..

[26] Ferrocino I., Ponzo V., Gambino R., Zarovska A., Leone F., Monzeglio C., Goitre I., Rosato R., Romano A., Grassi G. (2018). Changes in the gut microbiota composition during pregnancy in patients with gestational diabetes mellitus (GDM). Sci. Rep..

[27] Sugino K.Y., Hernandez T.L., Barbour L.A., Kofonow J.M., Frank D.N., Friedman J.E. (2022). A maternal higher-complex carbohydrate diet increases bifidobacteria and alters early life acquisition of the infant microbiome in women with gestational diabetes mellitus. Front. Endocrinol..

[28] Kocova M. (2020). Genetic spectrum of neonatal diabetes. Balkan J. Med. Genet..

[29] Greeley S.A.W., Tucker S.E., Naylor R.N., Bell G.I., Philipson L.H. (2010). Neonatal diabetes mellitus: A model for personalized medicine. Trends Endocrinol. Metab..

[30] Greeley S.A.W., Naylor R.N., Philipson L.H., Bell G.I. (2011). Neonatal diabetes: An expanding list of genes allows for improved diagnosis and treatment. Curr. Diabetes Rep..

[31] Naylor R.N., Greeley S.A.W., Bell G.I., Philipson L.H. (2011). Genetics and pathophysiology of neonatal diabetes mellitus. J. Diabetes Investig..

[32] Vaxillaire M., Bonnefond A., Froguel P. (2012). The lessons of early-onset monogenic diabetes for the understanding of diabetes pathogenesis. Best. Pract. Res. Clin. Endocrinol. Metab..

[33] Zübarioğlu A.U., Bülbül A., Uslu H.S. (2018). Neonatal diabetes mellitus. Sisli Etfal Hastan. Tip Bülteni.

[34] Dahl A., Kumar S. (2020). Recent Advances in Neonatal Diabetes. Diabetes Metab. Syndr. Obes..

[35] Zhang H., Colclough K., Gloyn A.L., Pollin T.I. (2021). Monogenic diabetes: A gateway to precision medicine in diabetes. J. Clin. Investig..

[36] Bak M., Boonen S.E., Dahl C., Hahnemann J.M.D., Mackay D.J.D.G., Tümer Z., Grønskov K., Temple I.K., Guldberg P., Tommerup N. (2016). Genome-wide DNA methylation analysis of transient neonatal diabetes type 1 patients with mutations in ZFP57. BMC Med. Genet..

[37] Mackay D.J.G., Temple I.K. (2010). Transient neonatal diabetes mellitus type 1. Am. J. Med. Genet. C Semin. Med. Genet..

[38] Akerman I., Maestro M.A., De Franco E., Grau V., Flanagan S., García-Hurtado J., Mittler G., Ravassard P., Piemonti L., Ellard S. (2021). Neonatal diabetes mutations disrupt a chromatin pioneering function that activates the human insulin gene. Cell Rep..

[39] Hammoud B., Greeley S.A.W. (2022). Growth and development in monogenic forms of neonatal diabetes. Curr. Opin. Endocrinol. Diabetes Obes..

[40] Johnson M.B., Patel K.A., De Franco E., Hagopian W., Killian M., McDonald T.J., Tree T.I.M., Domingo-Vila C., Hudson M., Hammersley S. (2020). Type 1 diabetes can present before the age of 6 months and is characterized by autoimmunity and rapid loss of beta cells. Diabetologia.

[41] Yan J., Yang H. (2014). Gestational diabetes mellitus, programing and epigenetics. J. Matern. Fetal Neonatal Med..

[42] Hjort L., Martino D., Grunnet L.G., Naeem H., Maksimovic J., Olsson A.H., Zhang C., Ling C., Olsen S.F., Saffery R. (2018). Gestational diabetes and maternal obesity are associated with epigenome-wide methylation changes in children. JCI Insight.

[43] Hjort L., Novakovic B., Grunnet L.G., Maple-Brown L., Damm P., Desoye G., Saffery R. (2019). Diabetes in pregnancy and epigenetic mechanisms-how the first 9 months from conception might affect the child’s epigenome and later risk of disease. Lancet Diabetes Endocrinol..

[44] Lu S., Wang J., Kakongoma N., Hua W., Xu J., Wang Y., He S., Gu H., Shi J., Hu W. (2022). DNA methylation and expression profiles of placenta and umbilical cord blood reveal the characteristics of gestational diabetes mellitus patients and offspring. Clin. Epigenetics.

[45] Ott R., Stupin J.H., Melchior K., Schellong K., Ziska T., Dudenhausen J.W., Henrich W., Rancourt R.C., Plagemann A. (2018). Alterations of adiponectin gene expression and DNA methylation in adipose tissues and blood cells are associated with gestational diabetes and neonatal outcome. Clin. Epigenetics.

[46] Kang J., Lee C.-N., Li H.-Y., Hsu K.-H., Lin S.-Y. (2017). Genome-wide DNA methylation variation in maternal and cord blood of gestational diabetes population. Diabetes Res. Clin. Pract..

[47] Ruchat S.-M., Houde A.-A., Voisin G., St-Pierre J., Perron P., Baillargeon J.-P., Gaudet D., Hivert M.-F., Brisson D., Bouchard L. (2013). Gestational diabetes mellitus epigenetically affects genes predominantly involved in metabolic diseases. Epigenetics.

[48] Winzell M.S., Ahrén B. (2007). Role of VIP and PACAP in islet function. Peptides.

[49] Soubeyrand S., Martinuk A., Naing T., Lau P., McPherson R. (2016). Role of Tribbles Pseudokinase 1 (TRIB1) in human hepatocyte metabolism. Biochim. Biophys. Acta.

[50] Popova P.V., Vasileva L.B., Tkachuk A.S., Puzanov M.V., Bolotko Y.A., Pustozerov E.A., Gerasimov A.S., Zazerskaya I.E., Li O.A., Vasilyeva E.Y. (2018). Association of tribbles homologue 1 gene expression in human umbilical vein endothelial cells with duration of intrauterine exposure to hyperglycaemia. Genet. Res..

[51] Sukarieh R., Joseph R., Leow S.C., Li Y., Löffler M., Aris I.M., Tan J.H., Teh A.L., Chen L., Holbrook J.D. (2014). Molecular pathways reflecting poor intrauterine growth are found in Wharton’s jelly-derived mesenchymal stem cells. Hum. Reprod..

[52] Wang G., Liang R., Liu T., Wang L., Zou J., Liu N., Liu Y., Cai X., Liu Y., Ding X. (2019). Opposing effects of IL-1β/COX-2/PGE2 pathway loop on islets in type 2 diabetes mellitus. Endocr. J..

[53] Xu P., Dong S., Wu L., Bai Y., Bi X., Li Y., Shu C. (2023). Maternal and placental DNA methylation changes associated with the pathogenesis of gestational diabetes mellitus. Nutrients.

[54] Hjort L., Novakovic B., Cvitic S., Saffery R., Damm P., Desoye G. (2022). Placental DNA methylation in pregnancies complicated by maternal diabetes and/or obesity: State of the art and research gaps. Epigenetics.

[55] Xie X., Gao H., Zeng W., Chen S., Feng L., Deng D., Qiao F.-Y., Liao L., McCormick K., Ning Q. (2015). Placental DNA methylation of peroxisome-proliferator-activated receptor-γ co-activator-1α promoter is associated with maternal gestational glucose level. Clin. Sci..

[56] Ruschke K., Fishbein L., Dietrich A., Klöting N., Tönjes A., Oberbach A., Fasshauer M., Jenkner J., Schön M.R., Stumvoll M. (2010). Gene expression of PPARgamma and PGC-1alpha in human omental and subcutaneous adipose tissues is related to insulin resistance markers and mediates beneficial effects of physical training. Eur. J. Endocrinol..

[57] Côté S., Gagné-Ouellet V., Guay S.-P., Allard C., Houde A.-A., Perron P., Baillargeon J.-P., Gaudet D., Guérin R., Brisson D. (2016). PPARGC1α gene DNA methylation variations in human placenta mediate the link between maternal hyperglycemia and leptin levels in newborns. Clin. Epigenetics.

[58] Gao Y., She R., Sha W. (2017). Gestational diabetes mellitus is associated with decreased adipose and placenta peroxisome proliferator-activator receptor γ expression in a Chinese population. Oncotarget.

[59] Wang L., Fan H., Zhou L., Wu Y., Lu H., Luo J. (2018). Altered expression of PGC-1α and PDX1 and their methylation status are associated with fetal glucose metabolism in gestational diabetes mellitus. Biochem. Biophys. Res. Commun..

[60] Mohammadi A., Higazy R., Gauda E.B. (2022). PGC-1α activity and mitochondrial dysfunction in preterm infants. Front. Physiol..

[61] Gupta D., Leahy A.A., Monga N., Peshavaria M., Jetton T.L., Leahy J.L. (2013). Peroxisome proliferator-activated receptor γ (PPARγ) and its target genes are downstream effectors of FoxO1 protein in islet β-cells: Mechanism of β-cell compensation and failure. J. Biol. Chem..

[62] El Hajj N., Pliushch G., Schneider E., Dittrich M., Müller T., Korenkov M., Aretz M., Zechner U., Lehnen H., Haaf T. (2013). Metabolic programming of MEST DNA methylation by intrauterine exposure to gestational diabetes mellitus. Diabetes.

[63] Weng X., Liu F., Zhang H., Kan M., Wang T., Dong M., Liu Y. (2018). Genome-wide DNA methylation profiling in infants born to gestational diabetes mellitus. Diabetes Res. Clin. Pract..

[64] Bentley L., Nakabayashi K., Monk D., Beechey C., Peters J., Birjandi Z., Khayat F.E., Patel M., Preece M.A., Stanier P. (2003). The imprinted region on human chromosome 7q32 extends to the carboxypeptidase A gene cluster: An imprinted candidate for Silver-Russell syndrome. J. Med. Genet..

[65] Sparago A., Cerrato F., Riccio A. (2018). Is ZFP57 binding to *H19/IGF2*:IG-DMR affected in Silver-Russell syndrome?. Clin. Epigenetics.

[66] Brereton R.E., Nickerson S.L., Woodward K.J., Edwards T., Sivamoorthy S., Walters F.R.V., Chabros V., Marchin V., Grumball T., Kennedy D. (2021). Further heterogeneity in Silver-Russell syndrome: PLAG1 deletion in association with a complex chromosomal rearrangement. Am. J. Med. Genet. A.

[67] Quilter C.R., Cooper W.N., Cliffe K.M., Skinner B.M., Prentice P.M., Nelson L., Bauer J., Ong K.K., Constância M., Lowe W.L. (2014). Impact on offspring methylation patterns of maternal gestational diabetes mellitus and intrauterine growth restraint suggest common genes and pathways linked to subsequent type 2 diabetes risk. FASEB J..

[68] Ng M.C.Y., Lam V.K.L., Tam C.H.T., Chan A.W.H., So W.-Y., Ma R.C.W., Zee B.C.Y., Waye M.M.Y., Mak W.W., Hu C. (2010). Association of the POU class 2 homeobox 1 gene (POU2F1) with susceptibility to Type 2 diabetes in Chinese populations. Diabet. Med..

[69] Wang P., Jin T. (2010). Hydrogen peroxide stimulates nuclear import of the POU homeodomain protein Oct-1 and its repressive effect on the expression of Cdx-2. BMC Cell Biol..

[70] Primo-Parmo S.L., Sorenson R.C., Teiber J., Du B.N.L. (1996). The human serum paraoxonase/arylesterase gene (PON1) is one member of a multigene family. Genomics.

[71] León I., Roldán S.H., José Rodrigo M., Rodríguez M.L., Fisher J., Mitchell C., Lage-Castellanos A. (2022). The shared mother-child epigenetic signature of neglect is related to maternal adverse events. Front. Physiol..

[72] Pappa K.I., Gazouli M., Anastasiou E., Loutradis D., Anagnou N.P. (2017). The Q192R polymorphism of the paraoxonase-1 (PON1) gene is associated with susceptibility to gestational diabetes mellitus in the Greek population. Gynecol. Endocrinol..

[73] Zhou M., Liu X.-H., Liu Q.-Q., Chen M., Bai H., Jiang C.-Y., Guan L.-B., Fan P. (2021). Lactonase activity and status of paraoxonase 1 and oxidative stress in neonates of women with gestational diabetes mellitus. Pediatr. Res..

[74] Gugliucci A., Numaguchi M., Caccavello R., Kimura S. (2014). Paraoxonase 1 lactonase activity and distribution in the HDL subclasses in the cord blood. Redox Rep..

[75] Cheng X., Siow R.C.M., Mann G.E. (2011). Impaired redox signaling and antioxidant gene expression in endothelial cells in diabetes: A role for mitochondria and the nuclear factor-E2-related factor 2-Kelch-like ECH-associated protein 1 defense pathway. Antioxid. Redox Signal..

[76] Cheng X., Chapple S.J., Patel B., Puszyk W., Sugden D., Yin X., Mayr M., Siow R.C.M., Mann G.E. (2013). Gestational diabetes mellitus impairs Nrf2-mediated adaptive antioxidant defenses and redox signaling in fetal endothelial cells in utero. Diabetes.

[77] Baumel-Alterzon S., Scott D.K. (2022). Regulation of Pdx1 by oxidative stress and Nrf2 in pancreatic beta-cells. Front. Endocrinol..

[78] Tobi E.W., Juvinao-Quintero D.L., Ronkainen J., Ott R., Alfano R., Canouil M., Geurtsen M.L., Khamis A., Küpers L.K., Lim I.Y. (2022). Maternal glycemic dysregulation during pregnancy and neonatal blood DNA methylation: Meta-analyses of epigenome-wide association studies. Diabetes Care.

[79] Wondafrash D.Z., Nire’a A.T., Tafere G.G., Desta D.M., Berhe D.A., Zewdie K.A. (2020). Thioredoxin-interacting protein as a novel potential therapeutic target in diabetes mellitus and its underlying complications. Diabetes Metab. Syndr. Obes..

[80] Basnet R., Basnet T.B., Basnet B.B., Khadka S. (2022). Overview on thioredoxin-interacting protein (TXNIP): A potential target for diabetes intervention. Curr. Drug. Targets.

[81] Sarina, Li D.F., Feng Z.Q., Du J., Zhao W.H., Huang N., Jia J.C., Wu Z.Y., Alamusi, Wang Y.Y. (2019). Mechanism of placenta damage in gestational diabetes mellitus by investigating TXNIP of patient samples and gene functional research in cell line. Diabetes Ther..

[82] Pasternak Y., Ohana M., Biron-Shental T., Cohen-Hagai K., Benchetrit S., Zitman-Gal T. (2020). Thioredoxin, thioredoxin interacting protein and transducer and activator of transcription 3 in gestational diabetes. Mol. Biol. Rep..

[83] Velayos T., Martínez R., Alonso M., Garcia-Etxebarria K., Aguayo A., Camarero C., Urrutia I., Martínez de LaPiscina I., Barrio R., Santin I. (2017). An activating mutation in *STAT3* results in neonatal diabetes through reduced insulin synthesis. Diabetes.

[84] Mokhtari P., Metos J., Babu P.V.A. (2021). Impact of type 1 diabetes on the composition and functional potential of gut microbiome in children and adolescents: Possible mechanisms, current knowledge, and challenges. Gut Microbes.

[85] Del Chierico F., Rapini N., Deodati A., Matteoli M.C., Cianfarani S., Putignani L. (2022). Pathophysiology of type 1 diabetes and gut microbiota role. Int. J. Mol. Sci..

[86] Yang Q., Ouyang J., Sun F., Yang J. (2020). Short-chain fatty acids: A soldier fighting against inflammation and protecting from tumorigenesis in people with diabetes. Front. Immunol..

[87] Yi C., Sun W., Ding L., Yan M., Sun C., Qiu C., Wang D., Wu L. (2022). Short-chain fatty acids weaken ox-LDL-induced cell inflammatory injury by inhibiting the NLRP3/Caspase-1 pathway and affecting cellular metabolism in THP-1 cells. Molecules.

[88] Feng Y., Wang Y., Wang P., Huang Y., Wang F. (2018). Short-chain fatty acids manifest stimulative and protective effects on intestinal barrier function through the inhibition of NLRP3 inflammasome and autophagy. Cell Physiol. Biochem..

[89] Yaku K., Enami Y., Kurajyo C., Matsui-Yuasa I., Konishi Y., Kojima-Yuasa A. (2012). The enhancement of phase 2 enzyme activities by sodium butyrate in normal intestinal epithelial cells is associated with Nrf2 and p53. Mol. Cell Biochem..

[90] Milani C., Mancabelli L., Lugli G.A., Duranti S., Turroni F., Ferrario C., Mangifesta M., Viappiani A., Ferretti P., Gorfer V. (2015). Exploring vertical transmission of Bifidobacteria from mother to child. Appl. Environ. Microbiol..

[91] Asnicar F., Manara F., Zolfo M., Truong D.T., Scholz M., Armanini F., Ferretti P., Gorfer V., Pedrotti A., Tett A. (2017). Studying vertical microbiome transmission from mothers to infants by strain-level metagenomic profiling. mSystems.

[92] Ferretti P., Pasolli E., Tett A., Asnicar F., Gorfer V., Fedi S., Armanini F., Truong D.T., Manara S., Zolfo M. (2018). Mother-to-infant microbial transmission from different body sites shapes the developing infant gut microbiome. Cell Host Microbe..

[93] Koo H., McFarland B.C., Hakim J.A., Crossman D.K., Crowley M.R., Rodriguez J.M., Benveniste E.N., Morrow C.D. (2020). An individualized mosaic of maternal microbial strains is transmitted to the infant gut microbial community. R. Soc. Open. Sci..

[94] Ponzo V., Ferrocino I., Zarovska A., Amenta M.B., Leone F., Monzeglio C., Rosato R., Pellegrini M., Gambino R., Cassader M. (2019). The microbiota composition of the offspring of patients with gestational diabetes mellitus (GDM). PLoS ONE.

[95] Kuang Y.-S., Lu J.-H., Li S.-H., Li J.-H., Yuan M.-Y., He J.-R., Chen N.-N., Xiao W.-Q., Shen S.-Y., Qiu L. (2017). Connections between the human gut microbiome and gestational diabetes mellitus. Gigascience.

[96] Soderborg T.K., Carpenter C.M., Janssen R.C., Weir T.L., Robertson C.E., Ir D., Young B.E., Krebs N.F., Hernandez T.L., Barbour L.A. (2020). Gestational diabetes is uniquely associated with altered early seeding of the infant gut microbiota. Front. Endocrinol..

[97] Gopalsamy G., Mortimer E., Greenfield P., Bird A.R., Young G.P., Christophersen C.T. (2019). Resistant starch is actively fermented by infant faecal microbiota and increases microbial diversity. Nutrients.

[98] Nurmi J.T., Puolakkainen P.A., Rautonen N.E. (2005). *Bifidobacterium lactis* sp. 420 up-regulates cyclooxygenase (Cox)-1 and down-regulates Cox-2 gene expression in a Caco-2 cell culture model. Nutr. Cancer.

[99] Kawabata K., Baba N., Sakano T., Hamano Y., Taira S., Tamura A., Baba S., Natsume M., Ishii T., Murakami S. (2018). Functional properties of anti-inflammatory substances from quercetin-treated *Bifidobacterium adolescentis*. Biosci. Biotechnol. Biochem..

[100] Jost T., Lacroix C., Braegger C.P., Rochat F., Chassard C. (2014). Vertical mother-neonate transfer of maternal gut bacteria via breastfeeding. Environ. Microbiol..

[101] Li X., Yu D., Wang Y., Yuan H., Ning X., Rui B., Lei Z., Yuan J., Yan J., Li M. (2021). The intestinal dysbiosis of mothers with gestational diabetes mellitus (GDM) and its impact on the gut microbiota of their newborns. Can. J. Infect. Dis. Med. Microbiol..

[102] Arif S., Moore F., Marks K., Bouckenooghe T., Dayan C.M., Planas R., Vives-Pi M., Powrie J., Tree T., Marchetti O. (2011). Peripheral and islet interleukin-17 pathway activation characterizes human autoimmune diabetes and promotes cytokine-mediated β-cell death. Diabetes.

[103] Nepelska M., de Wouters T., Jacouton E., Béguet-Crespel F., Lapaque N., Doré J., Arulampalam V., Blottière H.M. (2017). Commensal gut bacteria modulate phosphorylation-dependent PPARγ transcriptional activity in human intestinal epithelial cells. Sci. Rep..

[104] Bhagwat A., Annapure U.S. (2019). Maternal-neonatal transmission of *Enterococcus* strains during delivery. Beni-Suef Univ. J. Basic. Appl. Sci..

[105] Vitetta L., Coulson S., Thomsen M., Nguyen T., Hall S. (2017). Probiotics D-lactic acidosis, oxidative stress and strain specificity. Gut Microbes.

[106] Matsumoto Y., Ishii M., Hasegawa S., Sekimizu K. (2019). *Enterococcus faecalis* YM0831 suppresses sucrose-induced hyperglycemia in a silkworm model and in humans. Commun. Biol..

[107] Are A., Aronsson L., Wang S., Greicius G., Lee Y.K., Gustafsson J.-A., Pettersson S., Arulampalam V. (2008). Enterococcus faecalis from newborn babies regulate endogenous PPARgamma activity and IL-10 levels in colonic epithelial cells. Proc. Natl. Acad. Sci. USA.

[108] Maqsood R., Rodriguez R.R.C., Handley S.A., Ndao I.M., Tarr P.I., Warner B.B., Lim E.S., Holtz L.R. (2019). Discordant transmission of bacteria and viruses from mothers to babies at birth. Microbiome.

[109] Chen F., Gan Y., Li Y., He W., Wu W., Wang K., Li Q. (2021). Association of gestational diabetes mellitus with changes in gut microbiota composition at the species level. BMC Microbiol..

[110] Su Y., Wang H.K., Gan X.P., Chen L., Cao Y.N., Cheng D.C., Zhang D.Y., Liu W.Y., Li F.F., Xu X.M. (2021). Alterations of gut microbiota in gestational diabetes patients during the second trimester of pregnancy in the Shanghai Han population. J. Transl. Med..

[111] Song Z., Li S., Li R. (2022). An investigation into the correlation of intestinal flora with obesity and gestational diabetes mellitus. Comput. Math. Methods Med..

[112] Talford L.E., Crost E.H., Kavanaugh D., Juge N. (2005). Mucin glycan foraging in the human gut microbiome. Front. Genet..

[113] Glover J.S., Ticer T.D., Engevik M.A. (2022). Characterizing the mucin-degrading capacity of the human gut microbiota. Sci. Rep..

[114] Koh A., De Vadder F., Kovatcheva-datchary P., Backhed F. (2016). From dietary fiber to host physiology: Short-chain fatty acids as key bacterial metabolites. Cell.

[115] Leite A.Z., de Campos Rodrigues N., Gonzaga M.I., Paiolo J.C.C., Arantes de Souza C., Stefanutto N.A.V., Omori W.P., Guariz Pinheiro D., Brisotti J.L., Junior E.M. (2017). Detection of increased plasma interleukin-6 levels and prevalence of *Prevotella copri* and *Bacteroides vulgatus* in the feces of type 2 diabetes patients. Front. Immunol..

[116] Jeon J.I., Choi J.H., Lee K.H., Kim J.M. (2020). *Bacteroides fragilis* enterotoxin induces sulfiredoxin-1 expression in intestinal epithelial cell lines through a mitogen-activated protein kinases- and Nrf2-dependent pathway, leading to the suppression of apoptosis. Int. J. Mol. Sci..

[117] David L.A., Maurice C.F., Carmody R.N., Gootenberg D.B., Button J.E., Wolfe B.E., Ling A.V., Devlin A.S., Varma Y., Fischbach M.A. (2014). Diet rapidly and reproducibly alters the human gut microbiome. Nature.

[118] Crusell M.K.W., Hansen T.H., Nielsen T., Allin K.H., Rühlemann M.C., Damm P., Vestergaard H., Rørbye C., Jørgensen N.R., Christiansen O.B. (2018). Gestational diabetes is associated with change in the gut microbiota composition in third trimester of pregnancy and postpartum. Microbiome.

[119] Wu Y., Bible P.W., Long S., Ming W.-K., Ding W., Long Y., Wen X., Li X., Deng X., Deng Y. (2020). Metagenomic analysis reveals gestational diabetes mellitus-related microbial regulators of glucose tolerance. Acta Diabetol..

[120] Gavin P.G., Mullaney J.A., Loo D., Lê Cao K.-A., Gottlieb P.A., Hill M.M., Zipris D., Hamilton-Williams E.E. (2018). Intestinal metaproteomics reveals host-microbiota interactions in subjects at risk for type 1 diabetes. Diabetes Care.

[121] d’Hennezel E., Abubucker S., Murphy L.O., Cullen T.W. (2017). Total lipopolysaccharide from the human gut microbiome silences toll-like receptor signaling. mSystems.

[122] Groh I.A.M., Riva A., Braun D., Sutherland H.G., Williams O., Bakuradze T., Pahlke G., Richling E., Haupt L.M., Griffiths L.R. (2020). Long-term consumption of anthocyanin-rich fruit juice: Impact on gut microbiota and antioxidant markers in lymphocytes of healthy males. Antioxidants.

[123] Hernandez-Sanabria E., Heiremans E., Arroyo M.C., Props R., Leclercq L., Snoeys J., Van de Wiele T. (2020). Short-term supplementation of celecoxib-shifted butyrate production on a simulated model of the gut microbial ecosystem and ameliorated in vitro inflammation. NPJ Biofilms Microbiomes.

[124] Pinto Y., Frishman S., Turjeman S., Eshel A., Nuriel-Ohayon M., Shrossel O., Ziv O., Walters W., Parsonnet J., Ley C. (2023). Gestational diabetes is driven by microbiota-induced inflammation months before diagnosis. Gut.

[125] Larsen J.M. (2017). The immune response to *Prevotella* bacteria in chronic inflammatory disease. Immunology.

[126] Pedersen H.K., Gudmundsdottir V., Nielsen H.B., Hyotylainen T., Nielsen T., Jensen B.A.H., Forslund K., Hildebrand F., Prifti E., Falony G. (2016). Human gut microbes impact host serum metabolome and insulin sensitivity. Nature.

[127] Tarallo S., Ferrero G., De Filippis F., Francavilla A., Pasolli E., Panero V., Cordero F., Segata N., Grioni S., Pensa R.G. (2022). Stool microRNA profiles reflect different dietary and gut microbiome patterns in healthy individuals. Gut.

[128] Linna-Kuosmanen S., Bosch V.T., Moreau P.R., Bouvy-Liivrand M., Niskanen H., Kansanen E., Kivelä A., Hartikainen J., Hippeläinen M., Kokki H. (2021). NRF2 is a key regulator of endothelial microRNA expression under proatherogenic stimuli. Cardiovasc. Res..

[129] Davin-Regli A., Pagès J.-M. (2015). *Enterobacter aerogenes* and *Enterobacter cloacae*; versatile bacterial pathogens confronting antibiotic treatment. Front. Microbiol..

[130] Scheithauer T.P.M., Herrema H., Yu H., Bakker G.J., Winkelmeijer M., Soukhatcheva G., Dai D., Ma C., Havik S.R., Balvers M. (2022). Gut-derived bacterial flagellin induces beta-cell inflammation and dysfunction. Gut Microbes..

